# Surface Engineering of *Escherichia coli* to Display Its Phytase (AppA) and Functional Analysis of Enzyme Activities

**DOI:** 10.3390/cimb46040215

**Published:** 2024-04-17

**Authors:** Patricia L. A. Muñoz-Muñoz, Celina Terán-Ramírez, Rosa E. Mares-Alejandre, Ariana B. Márquez-González, Pablo A. Madero-Ayala, Samuel G. Meléndez-López, Marco A. Ramos-Ibarra

**Affiliations:** 1 Biotechnology and Biosciences Research Group, School of Chemical Sciences and Engineering, Autonomous University of Baja California, Tijuana 22390, BCN, Mexico; lilian.munoz.munoz@uabc.edu.mx (P.L.A.M.-M.); celina.teran@ibt.unam.mx (C.T.-R.); rmares@uabc.edu.mx (R.E.M.-A.); arianamg@ad.unc.edu (A.B.M.-G.); pablo.madero@uabc.edu.mx (P.A.M.-A.); samuelmelendez@uabc.edu.mx (S.G.M.-L.); 2 Biochemical Sciences Graduate Program (Doctorate Studies), National Autonomous University of Mexico, Cuernavaca 62210, MOR, Mexico; 3 Biological and Biomedical Sciences Graduate Program (Doctorate Studies), University of North Carolina, Chapel Hill, NC 27599, USA; 4 Science and Engineering Graduate Program (Doctorate Studies), Autonomous University of Baja California, Tijuana 22390, BCN, Mexico

**Keywords:** *Escherichia coli*, acid phosphatase/phytase, bacterial surface display, outer membrane-linked biocatalyst, feed additive

## Abstract

*Escherichia coli* phytase (AppA) is widely used as an exogenous enzyme in monogastric animal feed mainly because of its ability to degrade phytic acid or its salt (phytate), a natural source of phosphorus. Currently, successful recombinant production of soluble AppA has been achieved by gene overexpression using both bacterial and yeast systems. However, some methods for the biomembrane immobilization of phytases (including AppA), such as surface display on yeast cells and bacterial spores, have been investigated to avoid expensive enzyme purification processes. This study explored a homologous protein production approach for displaying AppA on the cell surface of *E. coli* by engineering its outer membrane (OM) for extracellular expression. Sodium dodecyl sulfate-polyacrylamide gel electrophoresis analysis of total bacterial lysates and immunofluorescence microscopy of non-permeabilized cells revealed protein expression, whereas activity assays using whole cells or OM fractions indicated functional enzyme display, as evidenced by consistent hydrolytic rates on typical substrates (i.e., p-nitrophenyl phosphate and phytic acid). Furthermore, the in vitro results obtained using a simple method to simulate the gastrointestinal tract of poultry suggest that the whole-cell biocatalyst has potential as a feed additive. Overall, our findings support the notion that biomembrane-immobilized enzymes are reliable for the hydrolysis of poorly digestible substrates relevant to animal nutrition.

## 1. Introduction

Surface display is a biotechnological method that involves the molecular engineering of cellular surfaces to immobilize peptides/proteins on the plasma membrane and display them in the extracellular space. This approach has been successful in displaying different biomolecules, from peptides to enzymes and antibodies, using bacteria (e.g., *Escherichia coli*, referred to as bacterial display) and yeast cells (e.g., *Saccharomyces cerevisiae* and *Pichia pastoris*, referred to as yeast display) as hosts [1,2,3,4]. In particular, peptides and proteins to be displayed must be fused to an anchoring motif (carrier) to ensure surface expression. Different carriers have been used for bacterial display, such as outer membrane proteins, lipoproteins, and autotransporters [5,6,7,8,9], whereas the most usual carriers for yeast display are glycosylphosphatidylinositol (GPI)-dependent cell wall proteins and the Aga1-Aga2 anchor system [6,10,11,12]. This biotechnology has various potential applications, including vaccine development, peptide library screening, metal bioadsorption, and bioconversion using whole-cell biocatalysts [1,6,13,14,15].

Phytases (EC 3.1.3.8, 3.1.3.26, and 3.1.3.72) belong to the histidine acid phosphatase (HAP) superfamily and catalyze the removal of orthophosphate from phytic acid or its salt (phytate), which is the primary phosphorus storage compound in seeds. Given that phytase activity is absent in the digestive tract of monogastric animals (e.g., poultry and pigs), phytate is not metabolized and is therefore excreted undegraded, contributing to phosphorus pollution in regions with intensive livestock production [16,17,18,19]. In addition, phytic acid is recognized as an anti-nutrient that can reduce the bioavailability of essential minerals (by chelating calcium, magnesium, zinc, and iron) [20,21,22]. Microbial phytases have been widely used as feed additives, resulting in increased phosphorus utilization and mineral availability, thus reducing the negative environmental impacts [23,24,25].

*E. coli* AppA is a periplasmic enzyme that exhibits both acid phosphatase and phytase activity [26]. Optimal phosphatase activity was observed at pH 2.5, using p-nitrophenyl phosphate (pNPP) as a synthetic substrate (K_M_ = 2.8 mM). Moreover, it can hydrolyze the 5’-β-phosphoryl residue of ppGpp (K_M_ = 1.8 mM) and the γ-phosphoryl residue of GTP (K_M_ = 0.35 mM). However, it cannot cleave most phosphomonoester substrates except fructose 1,6-bisphosphate and 2,3-bisphosphoglycerate (K_M_ = 5 mM) [27]. In contrast, the optimal phytase activity was observed at pH 4.5 (K_M_ = 0.63 mM on phytic acid). Moreover, it performs hydrolysis in a stereospecific manner through sequential phosphate removal [26,28,29]. Among the bacterial phytases, AppA exhibits the most consistent features and displays significant resistance to proteolysis under physiological conditions, supporting its putative application in monogastric animal nutrition [24,30,31].

To date, only a few phytases have been successfully displayed on cell surfaces, using yeast as the preferred host for membrane engineering and whole-cell biocatalysis. PhyA from *Aspergillus niger* (a fungal phytase) was displayed on both *S. cerevisiae* and *P. pastoris*, utilizing the C-terminus of yeast α-agglutinin as a carrier [32,33]. In contrast, Phy from *Citrobacter amalonaticus* (a bacterial phytase) was displayed on *P. pastoris* using yeast GCW61, a GPI-anchored cell-wall glycoprotein homolog, as the fusion partner [34]. These specific approaches resulted in engineered yeast cells displaying active phytases, which exhibited promising prospects as whole-cell biocatalysts, but with a chance to improve enzyme stability and activity. More recently, *E. coli* AppA was displayed on the surface of *Bacillus subtilis* spores using the outer coat protein CotG as a carrier [18]. Although this approach favored valuable features of displayed AppA (e.g., protease resistance, protein stability, and enzyme efficiency), using appropriate peptide linkers may improve its full operativity.

Diverse systems have been developed to display proteins on bacterial surfaces, such as those using either outer membrane protein A (OmpA) or adhesin involved in diffusible adhesion-I (AIDA-I) from *E. coli*, ice nucleation protein (INP) homologs from ina^+^ bacteria (e.g., *Pseudomonas syringae*), and protein A from *Staphylococcus aureus* as anchoring motifs [3,6,7]. Among these, the Lpp-OmpA fusion remains the most popular [35,36] and has been successfully used to display a wide range of active enzymes, including β-lactamases and lipases, on the surface of *E. coli* [37,38]. The typical fusion comprises the N-terminal sequence (Met1 to Gln29) of the major lipoprotein (Lpp) and a transmembrane region of OmpA spanning five β-sheets (β3-β7: Ans67 to Asn180). Two major features support its pertinence (after proper signal processing and membrane embedding): the N-terminal residue (Lpp: Cys21) binds to OM lipids [39], and the C-terminal stretch (OmpA: Trp164 to Asn180) extends toward the extracellular space [37,40].

In a previous study, *E. coli* was successfully engineered to display the catalytic domain of EhCHT1 (an amoebic chitinase) on the bacterial surface using the eLpp-OmpA variant, resulting in a whole-cell (WC) biocatalyst with functional endochitinase activity [41]. The eLpp-OmpA variant includes an elongated transmembrane region of OmpA, spanning β1–β7 sheets (Ala22 to Asn180), which structurally resembles the native conformation and favors β-barrel stability [42,43]. To our knowledge, the display of *E. coli* phytase on whole-cell bacterial surfaces has not been investigated previously. Therefore, this study aimed to fuse eLpp-OmpA with AppA to produce a microbial WC biocatalyst with phytase activity (MWCB-phytase). In addition to the advantages associated with the carrier and protein passenger (described above), MWCB-phytase is environmentally friendly and promises competitive reaction rates. Moreover, it avoids cell lysis and enzyme purification techniques, which are costly, time-consuming, and often difficult to scale-up. Here, we report the surface engineering of *E. coli* to display its phytase using a homologous expression approach. The results of different analytical assays employing the engineered bacteria are also discussed.

## 2. Materials and Methods

### 2.1. Materials

Luria-Bertani (LB) medium was obtained from Becton Dickinson and Co. (Franklin Lakes, NJ, USA). Nucleic acid purification kits and other reagents for PCR amplification were purchased from Qiagen (Germantown, MD, USA). Restriction endonucleases and other enzymes used for molecular cloning were acquired from New England Biolabs (Ipswich, MA, USA). Reagents for protein analysis were obtained from Bio-Rad (Hercules, CA, USA) and GE Healthcare Biosciences (Pittsburgh, PA, USA). All other biomaterials were purchased from Sigma-Aldrich (St. Louis, MO, USA).

### 2.2. Strains and Plasmids

**Figure 1:**
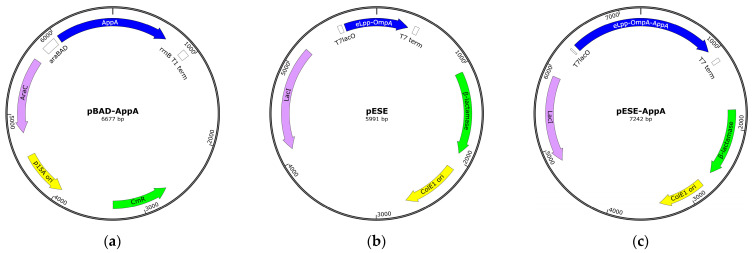
Schematic representation of pBAD-AppA, pESE, and pESE-AppA. (**a**) The pBAD-AppA plasmid is a pBAD33-based expression vector that encodes the periplasmic AppA protein (blue) under genetic regulation of the araBAD promoter rrnB T1 terminator. It includes other features, such as the arabinose promoter repressor (AraC, turquoise), chloramphenicol resistance gene (CmR, green), and bacterial origin of replication (p15A ori, yellow). (**b**) The pESE plasmid is a pET22b(+)-based expression vector that encodes the outer membrane (OM)-embedded eLpp-OmpA protein (blue) under genetic regulation of the T7lacO promoter and T7 terminator. It includes other features, such as the lactose promoter repressor (LacI, turquoise), ampicillin resistance gene (β-lactamase, green), and bacterial origin of replication (ColE1 ori, yellow). (**c**) The pESE-AppA plasmid is a pESE-based expression vector that encodes eLpp-OmpA-AppA (blue) under genetic control of the T7lacO promoter.

*E. coli* strains and plasmids used in the present study are listed in Table 1. Bacterial cells were cultivated in LB medium supplemented with antibiotics as required: ampicillin (0.15 mg/mL) and chloramphenicol (0.015 mg/mL). The ER2738 strain was employed for molecular cloning and plasmid propagation, whereas Lemo21(DE3) was used as the expression strain for surface display. The pBAD-AppA plasmid (Figure 1a) served as a template to amplify the sequence encoding mature AppA and the pESE plasmid (Figure 1b) was used as the backbone vector to display AppA on the bacterial surface.

### 2.3. PCR Amplification of AppA

The nucleotide sequence encoding mature AppA (Gln22-Leu432; UniProtKB entry ID: P07102) was amplified by PCR using *Pfu* DNA polymerase (Agilent, Santa Clara, CA, USA) and two synthetic primers, ECAPPAF2 (5′-caa caa gga tcc cag agt gag ccg gag ctg aag-3′) and BAD_RV (5′-tgg gac cac cgc gct act gcc-3′), acquired from Eurofins Genomics LLC (Louisville, KY, USA). The reaction mix included 20 pmol of each primer and 2 ng of the template (pBAD-AppA). Thermal cycling conditions involved an initial denaturation step at 94 °C for 2 min, followed by 35 cycles of exponential amplification (20 s at 94 °C, 20 s at 55 °C, and 90 s at 72 °C) and a final elongation step at 72 °C for 7 min. The PCR product (*Ec*AppA) was analyzed using standard agarose gel electrophoresis and purified using a QIAquick^®^ PCR Purification Kit (Qiagen, Germantown, MD, USA).

### 2.4. Construction of pESE-AppA

The pESE-AppA plasmid (Figure 1c) was constructed by inserting AppA (the PCR product) into pESE, using BamHI and HindIII cohesive ends as directional cloning sites. After proper endonucleolytic digestion, the restriction fragments (insert and vector) were joined using T4 DNA ligase (New England Biolabs, Ipswich, MA, USA), and the resulting product was used to transform competent bacteria (*E. coli* ER2738 strain). The recombinant plasmid was identified by colony PCR and purified using the QIAprep^®^ Spin Mini-prep Kit (Qiagen, Germantown, MD, USA). DNA sequencing confirmed the integrity of the AppA codons (i.e., insert).

### 2.5. Overexpression of the AppA Protein

*E. coli* Lemo21(DE3) harboring pESE-AppA was employed as a microbial system to express and display AppA. Stable transformants of the same strain harboring pET22b(+) or pESE were used as the internal controls. AppA overexpression was achieved in 5 or 10 mL batches of LB medium supplemented with antibiotics and inoculated (1:50) with fresh cultures. The standard conditions included initial cell proliferation for 2 h at 37 °C with constant shaking (300 rpm), induction with 0.5 mM IPTG (Sigma-Aldrich, St. Louis, MO, USA), and further proliferation for 16 h at 30 °C (300 rpm). The bacteria were harvested by centrifugation at 10,000× *g* for 10 min (10 °C) and washed twice with distilled water. After resuspension in the appropriate buffer, the optical density at 650 nm (OD_650_) was measured and used to normalize the bacterial cell number.

### 2.6. Analysis of AppA Expression and Display

#### 2.6.1. Sodium Dodecyl Sulfate-Polyacrylamide Gel Electrophoresis (SDS-PAGE)

Protein expression was determined using standard SDS-PAGE [46,47]. Bacteria from overexpression cultures (1.5 mL samples) were separated by centrifugation at 10,000 rpm for 2 min. The resulting pellets were resuspended in 0.2 mL lysis buffer (8 M urea in 100 mM Tris-HCl, pH 8.0) and disrupted for 5 min (3000 rpm). The resulting bacterial extract was thoroughly mixed with one volume of 2× Laemmli buffer and immediately denatured at 95 °C for 10 min. Aliquots (0.01 mL) were loaded onto a 13.5% polyacrylamide gel, and the proteins were separated under typical electrophoretic conditions.

#### 2.6.2. Immunofluorescence Microscopy (IFM)

Bacterial surface display was confirmed by IFM using an antibody, Fluorescein isothiocyanate (FITC)-conjugated, to detect Myc-tagged proteins. Engineered *E. coli* expressing eLpp-OmpA or eLpp-OmpA-AppA extend the Myc epitope towards the extracellular space. Cells from the overexpression cultures (OD_650_ = 2) were fixed for 90 min in 4% paraformaldehyde in PBS, washed twice with PBS, and blocked overnight with 1% bovine serum albumin (BSA) in PBS at 14 °C. The starting OD_650_ favored a homogeneous distribution of bacteria (250 ± 50 per field). Immunorecognition (5 µg/mL antibody) was performed for 90 min in the dark. After washing twice and resuspension in 0.2 mL PBS, samples (0.01 mL) of FITC-immunostained cells were mounted on slides and visualized using a Zeiss Axio Scope.A1 microscope connected to an AxioCam ICc-5 (Carl Zeiss Microscopy LLC, White Plains, NY, USA).

#### 2.6.3. Cell-based Acid Phosphatase Assay

A standard colorimetric assay was performed to determine whole-cell (WC)-linked acid phosphatase activity [48,49]. Typical reactions (0.25 mL) contained bacteria from overexpression cultures (OD_650_ = 0.3) and 10 mM p-nitrophenyl phosphate (pNPP) in 100 mM Gly-HCl (pH 2.5). The OD_650_ favored consistent kinetic rates. The reactions were incubated at 37 °C for 60 min with constant shaking (300 rpm) and then stopped by immediate mixing with 1.2 N NaOH (1.25 mL). After centrifugation at 16,000× *g* for 5 min, absorbance of the resulting supernatant was measured at 420 nm (A_420_). The p-nitrophenolate (pNP) concentration was estimated using the molar extinction coefficient (18,300 M^−1^ · cm^−1^). A cell-free reaction (uncatalyzed) was used as blank. When scatter from cell debris was detected, that is, a positive value for absorbance at 550 nm (A_550_), the A_420_ record was corrected by subtracting a common factor (1.75 × A_550_). WC-linked acid phosphatase activity was defined as the amount (nanomoles) of pNP released per minute per OD_650_ (nmol min^−1^ OD_650_^−1^).

### 2.7. Outer Membrane (OM)-Linked Activity Assays

Two colorimetric assays were performed to determine OM-linked acid phosphatase and phytase activities. The OM fraction was isolated according to a standard protocol [50], and a specific 100 mM reaction buffer was used to test each activity: Gly-HCl (pH 2.5) for acid phosphatase and acetate (pH 5.0) for phytase.

#### 2.7.1. OM Isolation

Bacteria from overexpression cultures (OD_650_ = 5) were suspended in 0.4 mL 0.1 M Tris-HCl (pH 8.0) and mixed well with 0.4 mL of SEL solution (40 mM sucrose; 0.4 mM EDTA, pH 8.0; 0.4 mg/mL lysozyme). The starting OD_650_ favored a reliable OM fraction yield. After incubation at room temperature (RT) for 10 min, the cell suspension was mixed thoroughly with 0.8 mL of TMDT buffer (2% Triton X-100, 10 mM MgCl_2_, 1 U/mL DNase, in 50 mM Tris-HCl, pH 8.0) and kept on ice for 30 min. Cell debris were removed by centrifugation at 1000× *g* for 5 min (10 °C), and the resulting supernatant was centrifuged at 16,000× *g* for 30 min (10 °C). The resulting pellet (OM fraction) was washed twice with distilled water and solubilized in the reaction buffer (0.1 mL), which was specific for each assay (see Section 2.7). Protein content was estimated using the Bradford colorimetric assay.

#### 2.7.2. Acid Phosphatase Assay

Standard reactions (0.25 mL) contained the OM fraction proteins (2 µg) and 10 mM pNPP. The reactions were incubated at 37 °C for 60 min with constant shaking (300 rpm) and then stopped by mixing with 1.2 N NaOH (1.25 mL). After centrifugation at 16,000× *g* for 5 min, the resulting supernatant (cleared) was used to determine pNP concentration, as described previously. A protein-free reaction (i.e., uncatalyzed) served as the blank. Biomembrane-linked phosphatase activity was defined as nanomoles of pNP released per minute per milligram of OM protein (nmol min^−1^ mg^−1^).

#### 2.7.3. Phytase Assay

Standard reactions (0.3 mL) contained the OM fraction proteins (2 µg) and 0.1 mM phytic acid. The reactions were incubated at 37 °C for 60 min with constant shaking (300 rpm) and then stopped by mixing with 0.3 mL of 5% trichloroacetic acid. After stabilization for 15 min and centrifugation at 16,000× *g* for 15 min (10 °C), Pi concentration in the clarified supernatant was determined using a malachite green (MG) colorimetric assay [51]. A protein-free reaction (i.e., uncatalyzed) served as the blank. Biomembrane-linked phytase activity was defined as nanomoles of Pi released per minute per milligram of OM protein (nmol min^−1^ mg^−1^).

#### 2.7.4. MG Colorimetric Assay

The MG reagent was prepared daily by blending 0.13% malachite green (in 3.1 M H_2_SO_4_), 7.8% ammonium molybdate, and 5.2% Tween-20 in a 20:5:1 ratio. After stabilization for 30 min, the MG reagent was centrifuged at 16,000× *g* for 10 min. The colorimetric detection of released Pi was performed by mixing 0.2 mL of the supernatant (from phytase activity assay) with 0.05 mL of MG reagent. The absorbance at 650 nm (A_650_) was measured immediately after 10 min of color development and was used to determine the Pi concentration through linear interpolation from a standard curve.

### 2.8. Simulation of Poultry Digestive Tract

A simple in vitro method was used to simulate phytic acid dephosphorylation in the GIT of poultry (crop, stomach, and small intestine) [52]. Unless otherwise described, all incubations were performed at 40 °C with constant shaking (100 rpm). Crop digestion: Different numbers of bacteria from the overexpression cultures (OD_650_ = 1, 2, 3, 5, 7.5, 10, and 15) were suspended in 6 mL acetate buffer (50 mM; pH 5.0) containing 1 mM phytic acid and incubated for 30 min. Stomach digestion: 0.52 mL pepsin (21 mg/mL in 50 mM acetate buffer, pH 3.0) and 0.28 mL HCl (1 N) were added to the crop digest and incubated for 45 min. Small intestine digestion: 0.65 mL of both pancreatin (7.4 mg/mL) and NaHCO_3_ (1 M) were added to the stomach digest and incubated for 60 min. Test samples (0.5 mL) from the GIT were incubated at 70 °C for 15 min, chilled for 1 min, acidified with 1 mL HCl (1 N), and shaken overnight at RT. The resulting suspensions were centrifuged at 10,000 rpm for 30 min (10 °C), and the cleared supernatants were used to determine the Pi concentration using the MG colorimetric assay. For simple validation, this approach was also employed to assess the phosphohydrolase function of MWCB-phytase in mimicked poultry feed; finely ground commercial cereal (Kellogg′s Corn Flakes) supplemented with phytic acid. The simulation was performed under similar conditions, with the following adaptations in crop digestion: overexpression bacteria (OD_650_ = 7.5) were suspended in 6 mL of acetate buffer containing phytic acid (0.83 mM) and ground cereal (1 g).

### 2.9. Data Analysis

All data were obtained from three independent experiments and are reported as mean ± standard deviation. GraphPad Prism^®^ version 4 for Windows (Boston, MA, USA) was used for all the statistical analyses. The nonlinear regression analysis was performed using a one-site binding model and the equation: Y = Ymax·X/(k + X), where X is the number of bacterial cells (OD_650_), Y is the concentration of Pi released (μM), Ymax is the maximum concentration of Pi released (extrapolated to a very high number of bacterial cells), and k represents an equilibrium constant. This equation also describes the activity of an enzyme, and the resulting plot is known as a rectangular hyperbola or a saturation curve.

## 3. Results and Discussion

### 3.1. Production of eLpp-OmpA-AppA and Display of AppA

#### 3.1.1. Construction of the Expression Plasmid, pESE-AppA

**Figure 2:**
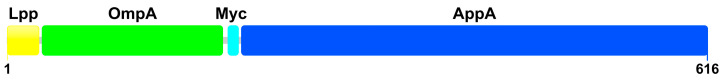
Schematic representation of eLpp-OmpA-AppA. Lpp (yellow), signal peptide plus nine residues of Lpp; OmpA (green), transmembrane region (β1-β7) of OmpA; Myc (cyan), cMyc epitope tag; AppA (blue), mature polypeptide of AppA.

The expression plasmid encoding the eLpp-OmpA-AppA protein (pESE-AppA, see Section 2.4) was constructed by subcloning the mature AppA sequence into pESE, a pET22b(+)-based vector that encodes eLpp-OmpA under control of the isopropyl-β-D-thiogalactopyranoside (IPTG)-inducible T7lacO promoter [41]. DNA sequencing confirmed the precise insertion of AppA codons (i.e., in-frame with those of eLpp-OmpA), which implies correct protein fusion (Figure 2) and the potential for gene expression under appropriate induction conditions. Furthermore, as a pET-based plasmid, pESE-AppA uses T7 RNA polymerase (T7RNAP) for high-level expression of cloned genes and constitutively expresses LacI, a lacO-binding protein that negatively regulates gene expression under non-induced conditions [53,54,55].

#### 3.1.2. Expression of eLpp-OmpA-AppA and Display of AppA

Recombinant protein production using a pET expression system requires *E. coli* cells containing the λDE3 lysogen, which carries the T7RNAP-encoding gene, with BL21(DE3) being the most widely used strain [55,56]. In this host, the IPTG-inducible lacUV5 promoter controls T7RNAP production, whereas expression of the recombinant protein (encoded by a gene located in a pET-based plasmid) is regulated by a T7RNAP-specific promoter [56]. Despite the advantages of BL21(DE3), it is unsuitable for producing recombinant proteins linked to growth problems (e.g., toxic or membrane proteins). Lemo21(DE3), an improved strain derived from BL21(DE3), has been developed to address these issues. This host harbors a plasmid encoding T7 lysozyme (T7Lys) under control of the rhamnose promoter [56,57,58]. In this host, the transcriptional activity of T7RNAP is controlled by the cellular abundance of T7Lys, a physiological inhibitor [59,60].

Results from our previous study showed that T7RNAP overload negatively affects the viability of BL21(DE3) cells harboring a pESE-derived plasmid (thus expressing the eLpp-OmpA carrier linked to the outer membrane), and basal co-expression with T7Lys eased this situation, as evidenced by the ordinary growth kinetics of Lemo21(DE3) cells subjected to similar experimental conditions [41]. Therefore, this improved strain was used as a host for pESE-AppA (i.e., the biofactory for eLpp-OmpA-AppA production) and as a cell source to assay biomembrane-linked enzyme activities.

**Figure 3:**
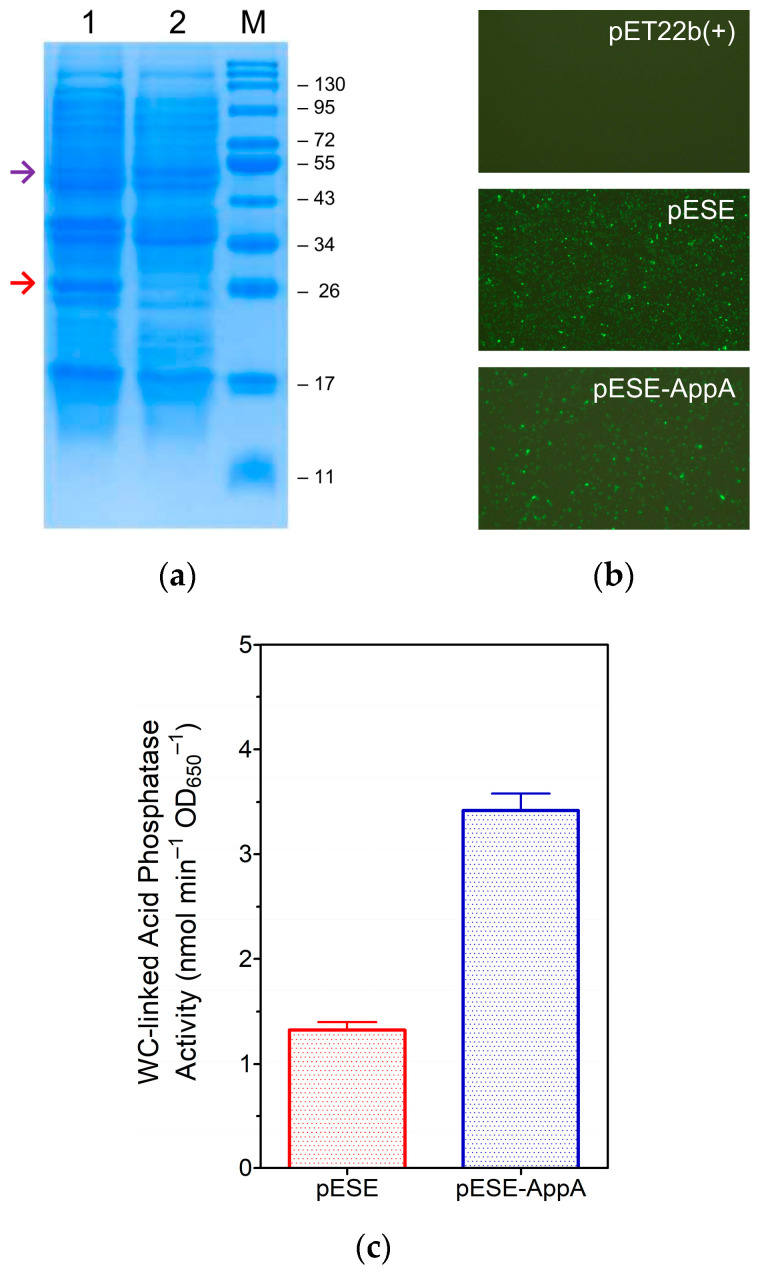
Analysis of protein expression in *E. coli* Lemo21(DE3) cells. (**a**) Electrophoretic separation of bacterial proteins by SDS-PAGE (12% gel stained with Coomassie Brilliant Blue). Lanes loaded with total extracts of cells harboring expression plasmids: 1: pESE; 2: pESE-AppA. Lane M, Protein MW standards (kDa). The colored arrows indicate the relative mobility of eLpp-OmpA (red, lane 1) and eLpp-OmpA-AppA (purple, lane 2). (**b**) Immunodetection of surface-displayed (Myc-tagged) proteins in non-permeabilized engineered bacteria using IFM (top-right label: expression plasmid). Note: Cells harboring the pET22b(+) plasmid served as controls to normalize the background (lack of fluorescence). Micrographs at 630× magnification. (**c**) Quantitative analysis of the WC-linked acid phosphatase activity. Substrate (pNPP) hydrolysis by engineered bacteria (bar label: expression plasmid) under standard conditions.

*E. coli* Lemo21(DE3) cells harboring the pESE-AppA construct or an internal control plasmid, either pET22b(+) or pESE, were used to determine protein production using standard protocols. Sodium dodecyl sulfate-polyacrylamide gel electrophoresis (SDS-PAGE) analysis revealed the consistent expression of eLpp-OmpA and eLpp-OmpA-AppA, which appeared as strongly stained bands in the respective cell extracts (Figure 3a). Fluorescence microscopic visualization of non-permeabilized bacteria stained with a fluorescein isothiocyanate (FITC)-conjugated anti-Myc(tag) antibody confirmed the surface display, as the cells expressing eLpp-OmpA or eLpp-OmpA-AppA, which extended the Myc epitope towards the extracellular space, emitted intense signals (Figure 3b). Furthermore, as a quantitative trait, bacteria expressing eLpp-OmpA-AppA protein showed a significant WC-linked acid phosphatase activity (*p* < 0.01), validating the functional display of AppA on the cell surface (Figure 3c).

#### 3.1.3. Analysis of OM-Linked AppA Activities

Although the above findings revealed that MWCB-phytase met expectations, one specific result caught our attention: bacteria harboring pESE, expressing only the carrier, showed a considerable degree of acid phosphatase activity (Figure 3c). This observation suggests that eLpp-OmpA overexpression affects outer membrane permeability, favoring both the influx of pNPP (substrate) into the periplasm and its availability for hydrolysis by native AppA, resulting in a detectable increase in the concentration of the leaving group (p-nitrophenolate, pNP). This notion is supported by the fact that OmpA acts as a low-permeability porin, allowing slow entry of small solutes [60]. Moreover, a pioneering study demonstrated that displaying β-lactamase (using Lpp-OmpA as a carrier) causes increased permeability, whereas no significant leakage of periplasmic proteins occurs [40]. Therefore, it is reasonable to assume that these changes in permeability are inevitable and represent a consequence of the large proteins displayed in *E. coli*.

**Figure 4:**
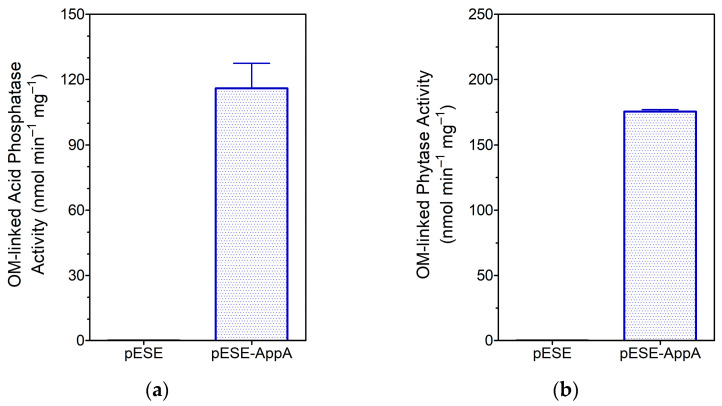
Functional analysis of AppA displayed on the surface of *E. coli* Lemo21(DE3). Quantitative detection of OM-linked activities: (**a**) acid phosphatase and (**b**) phytase. Substrate hydrolysis under standard conditions using OM fractions isolated from engineered bacteria (bar label: expression plasmid).

Supplementary analysis of the catalytic function of cell surface-displayed AppA by assaying acid phosphatase and phytase on OM fractions (i.e., free of activity associated with the periplasmic enzyme) corroborated our hypothesis. The OM fractions isolated from cells expressing eLpp-OmpA-AppA showed significant acid phosphatase and phytase activity (*p* < 0.001), whereas the equivalent fractions from cells expressing eLpp-OmpA showed no activity (Figure 4).

Although this result supports our goal, it is worth noting that the difference between the background and surface-linked activities (Figure 3c) can be improved because the Lemo21(DE3) cell system allows rhamnose-induced modulation of T7Lys, which in turn inhibits T7RNAP [56,57]. Therefore, using this approach, it is possible to regulate eLpp-OmpA-AppA expression, thus reducing OM permeability and likely resulting in a low phytate influx.

### 3.2. Evaluation of the WC Biocatalyst as a Poultry Feed Additive

On the optimistic side, the occurrence of background activity can be regarded as a positive attribute for the potential application of MWCB-phytase (poultry feed additive), as the cooperative action of both surface-displayed and native/soluble AppA benefits the biochemical goal, i.e., the enzyme-assisted dephosphorylation of phytate, thus improving the availability of this poorly digestible compound.

**Figure 5:**
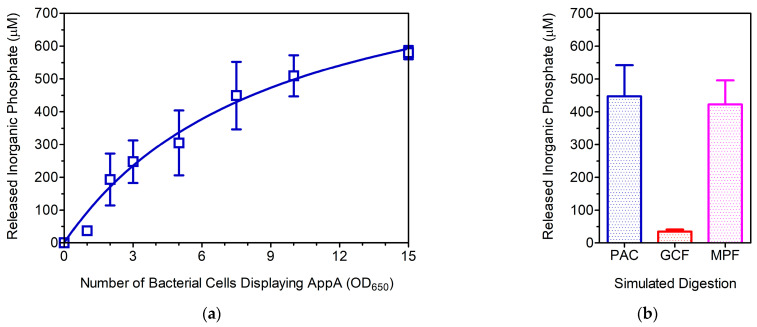
Functional analysis of MWCB-phytase under simulated poultry GIT conditions. (**a**) Effect of total bacterial number (OD_650_) on the dephosphorylation of phytic acid (presented as micromolar concentrations of released inorganic phosphate). The data (n = 3) were analyzed using a typical nonlinear method and fitted to a one-site binding model (r^2^ = 0.906). (**b**) Functional assessment of MWCB-phytase as a feed additive. Simulated digestion: phytic acid (PAC, blue), ground corn flakes (GCF, red), and mimicked poultry feed (MFP, magenta).

A simple in vitro approach was employed to investigate the effect of MWCB-phytase on phytic acid dephosphorylation in the gastrointestinal tract (GIT) of poultry. A starting volume of 6 mL favored homogeneous distribution of biomembrane-linked phytase. As expected, the dose-response analysis depicted a hyperbolic trend that fitted a one-site binding model (Figure 5a). The overall outcome of phytic acid dephosphorylation upon completion of the GIT course revealed that an equivalent cell number of 9.2 ± 2.5 OD_650_ reached half the predicted maximum concentration of released inorganic phosphate (Pi) (954 ± 139 μM). Further analysis using mimicked poultry feed, a finely ground commercial corn cereal supplemented with phytic acid, supports the potential application of AppA-displaying (engineered) bacteria. Under similar conditions, the resulting Pi concentrations were similar between digestions containing either mimicked feed or phytic acid alone (Figure 5b). Moreover, the control digestion (containing only commercial cereal) showed a marginal Pi concentration, which was regarded as a non-significant contributor to the final result (*p* < 0.01), thus validating the latter observation.

### 3.3. Is MWCB-Phytase Promising for Monogastric Animal Feed?

Bacterial display is a technology for expressing proteins linked to a biological surface that offers various advantages, such as high yield and productivity [61,62]. Several proteins have been displayed on bacteria, which have exhibited potential as cell-based systems for applications in biotechnology and biomedicine, ranging from whole-cell biosensors and biocatalysts to cell-based platforms for antibody screening [63,64,65,66,67,68,69].

The gastrointestinal tract of monogastric animals is influenced by natural factors such as microbiota diversity and environmental pH fluctuations [20,69], which contribute to complex physiological conditions that can affect the phytic acid degradation. Therefore, MWCB-phytase may be suitable for animal feed applications, as *E. coli* (a commensal in the GIT of numerous mammals, including monogastric animals [70,71]) has evolved to provide stability to surface proteins under complex physiological conditions [72,73,74].

In this study, *E. coli* Lemo21(DE3) cells were engineered to surface-display the AppA enzyme, which promises immediate use after harvesting and easy reuse after storage. At the laboratory scale, these bacteria can dephosphorylate phytic acid under simulated poultry GIT conditions, making them alternative biocatalysts (i.e., MWCB-phytase) for use as feed additives. Furthermore, this bacterial system offers the prospect of generating functionalized outer membrane vesicles ready for biocatalytic applications (such as the production of OMV-phytase) [75]. However, further studies are required to translate this system to animal nutrition.

The use of genetically engineered bacteria (GEB) has resulted in significant advances in food production (i.e., increasing efficiency, reducing waste, and optimizing resources), allowing beneficial innovations. However, international regulatory agencies must review the safety of GEB and the food goods produced to ensure that both microorganisms and the resulting products are safe for the environment, as well as human and animal health [76,77,78].

## 4. Conclusions

The eLpp-OmpA system is a reliable carrier for generating MWCB-phytase, and the fusion protein (eLpp-OmpA-AppA) was successfully embedded in the outer membrane of *E. coli* Lemo21(DE3), resulting in engineered bacteria with active expression and display of functional AppA. MWCB-phytase catalyzes efficient dephosphorylation of phytic acid under simulated poultry GIT conditions (in vitro). However, the biophysical and biochemical properties of AppA should be extensively assessed to determine whether membrane anchoring affects enzyme function for repeated use. Moreover, novel protein engineering strategies (e.g., AI-assisted) could help preserve and improve protein stability and catalytic efficiency under harsh production, transport, and storage conditions.

Overall, this study supports the application of biomembrane-linked phytase as a feed additive for proper phytate digestion, thereby improving its bioavailability and reducing its negative environmental impact.

## Figures and Tables

**Table 1:** *E. coli* strains and bacterial plasmids used throughout this study.

Strain	Genotype	Source
ER2728	F′ proA+B+ lacIq Δ(lacZ)M15 zzf::Tn10(Tet^R^)/fhuA2 glnV Δ(lac-proAB) thi-1 Δ(hsdS-mcrB)5	New England Biolabs
BL21(DE3)	fhuA2 [lon] ompT gal (λ DE3) [dcm] ∆hsdS λ DE3 = λ sBamHIo ∆EcoRI-B int::(lacI::PlacUV5::T7 gene1)i21 ∆nin5	Novagen
Lemo21(DE3)	BL21(DE3)/pLemo pLemo = pACYC184-PrhaBAD-lysY (Cam^R^)	New England Biolabs
MWC-22b	Lemo21(DE3)/pET22b(+)	This study
MWC-ESE	Lemo21(DE3)/pESE	This study
MWCB-phytase	Lemo21(DE3)/pESE-AppA	This study
Plasmid	Features	Source
pBAD33	araBAD promoter (arabinose regulation), p15A origin, Cam^R^	ATCC ^1^ [44]
pBAD-AppA	pBAD33-based, periplasmic expression of AppA	Lab Stock [45]
pET22b(+)	T7lacO promoter (lactose regulation), ColE1 origin, Amp^R^	Novagen
pESE	pET22b(+)-based, OM-linked expression of eLpp-OmpA	Lab Stock [41]
pESE-AppA	pESE-derived, OM-linked expression of eLpp-OmpA-AppA	This study

^1^ American type culture collection.

## Data Availability

This paper included all relevant data, excluding nucleotide and protein sequences (available elsewhere). The materials are available from the corresponding author upon request from qualified researchers without any undue reservation.
